# Antisense oligonucleotides targeting the progesterone receptor inhibit hormone-independent breast cancer growth in mice

**DOI:** 10.1186/bcr1345

**Published:** 2005-11-09

**Authors:** Caroline A Lamb, Luisa A Helguero, Sebastián Giulianelli, Rocío Soldati, Silvia I Vanzulli, Alfredo Molinolo, Claudia Lanari

**Affiliations:** 1 Instituto de Biología y Medicina Experimental (Consejo Nacional de Investigaciones Científicas y Técnicas (CONICET)), Buenos Aires, Argentina; 2 Oral and Pharyngeal Cancer Branch, National Institute of Dental and Craniofacial Research, National Institutes of Health, Bethesda, Maryland, USA

## Abstract

**Introduction:**

Previous data from our laboratory suggested that progesterone receptors (PRs) are involved in progestin-independent growth of mammary carcinomas. To investigate this possibility further, we studied the effects of PR antisense oligodeoxynucleotides (asPR) on *in vivo *tumor growth.

**Method:**

BALB/c mice with subcutaneous 25 mm^2 ^mammary carcinomas expressing estrogen receptor-α and PR were either injected intraperitoneally with 1 mg asPR every 24 or 12 hours for 5–10 days, or subcutaneously with RU 486 (6.5 mg/kg body weight) every 24 hours. Control mice received vehicle or scPR.

**Results:**

Significant inhibition of tumor growth as well as a significant decrease in bromodeoxyuridine uptake was observed in asPR-treated mice, which correlated with histological signs of regression and increased apoptosis. Mice treated with RU 486 experienced almost complete tumor regression. No differences were detected between vehicle-treated and scPR-treated mice. Anti-progestin-treated and asPR-treated mice were in a continuous estrous/meta-estrous state. Decreased phosphorylated extracellular signal-regulated kinase (ERK)1 and ERK2 levels and estrogen receptor-α expression were observed as late events in RU 486-treated and asPR-treated mice with regressing tumors.

**Conclusion:**

We demonstrate, for the first time, inhibition of tumor growth *in vivo *using asPR. Our results provide further evidence for a critical and hierarchical role of the PR pathway in mammary carcinomas.

## Introduction

The predominant role assigned to the estrogen receptor (ER)-α pathway as the key player in the origin and maintenance of the neoplastic phenotype in mammary cancer has been challenged by recent clinical [1-4] and experimental [5] findings, which suggest that progesterone receptors (PRs) play a similarly significant role. We and others have demonstrated in female mice that progesterone, as well as the synthetic progestin medroxyprogesterone acetate (MPA), can directly induce mammary carcinomas or act as a co-carcinogen [6-10]. We found clear differences in target cells or in the type of tumors induced by progesterone or MPA in mouse mammary gland [6], even though both agents are similarly able to promote hormone-dependent growth [11]. MPA induces mammary tumors that are quite similar to human breast cancers, specifically metastatic carcinomas of ductal histology, preceded by ductal pre-neoplastic lesions, which maintain high levels of ER-α and PRs and may progress through different stages of hormone responsiveness [12-14]. Progesterone, on the other hand, induces lobular mammary carcinomas, which lose hormone receptor expression after serial transplantations [6].

In previous reports we suggested that PRs are essential in the maintenance of the neoplastic phenotype of MPA-induced mammary carcinomas [14,15], even in tumors that have progressed to a hormone-independent phenotype [16]. Moreover, we also demonstrated that the PR pathway is also used by basic fibroblast growth factor to exert growth stimulatory effects [17]. Antisense oligodeoxynucleotides designed to block both PR isoforms (asPR) confirmed that PRs are involved in the basic fibroblast growth factor proliferative pathway in these cells [17]. This interaction between growth factors and PRs was recently confirmed by Labriola and coworkers [18], who demonstrated activation of PRs by mitogen-activated protein kinase (MAPK) using both our murine cells and T47D human cells; and by Qiu and coworkers [19], who demonstrated that activation of MAPK in human cells induced Ser294 phosphorylation and rapid nuclear translocation.

To further define the role played by the stimulatory effects of PRs in hormone-independent tumors within an experimental therapeutic scenario, we expanded our *in vitro *studies by blocking PRs with phosphorothiolated oligodeoxynucleotides to PR (i.e. asPR). Furthermore, we extended these findings to three different progestin-independent tumor lines in order to elucidate the involvement of PRs in the growth of such tumors. The goal of the present study was to investigate the *in vivo *effect of asPR on tumor growth.

## Materials and methods

### Animals

Two-month-old virgin female BALB/c mice (Instituto de Biología y Medicina Experimental Animal Facility, Buenos Aires, Argentina) were used. The animals were housed in groups of four per cage in an air-conditioned room at 20 ± 2°C under a 12-hour light/dark cycle, and had free access to food and tap water. Animal care and manipulation were in accordance with institutional guidelines and with the Guide for the Care and Use of Laboratory Animals [20].

### Tumors

Four different MPA-induced mammary ductal carcinomas were used, namely 59-2-HI, 32-2-HI, C7-2-HI and CC4-HI [13,21], which were maintained by serial subcutaneous implantation into BALB/c virgin female mice. These tumors belong to the group of responsive progestin-independent tumors; they do not need administration of exogenous hormones to grow but they regress when they are treated with estradiol (E_2_), RU 38486 (RU 486), or ZK 98299 (ZK 299) [22].

### Reagents

The reagents used in Western blots were purchased from Gibco BRL (New York, NY, USA). Methanol was purchased from Merck Química (Buenos Aires, Argentina). Molecular weight markers are Rainbow pre-stained molecular weight markers (Amersham Life Science, Buckinghamshire, UK). [^3^H]R5020 and [^3^H]R5020 were purchased from NEN (Boston, MA, USA) and KCl from Anedra (Buenos Aires, Argentina). Dithiothreitol, EDTA, sucrose, protease inhibitors, RU 486, and E_2 _were purchased from Sigma (St. Louis, MO, USA). ZK 299 was kindly provided by Schering (Berlin, Germany). Oligonucleotides were purchased from DNAgency (Malvern, PA, USA). They were designed to cover the translation initiation site of the target sequence. Synthetic, phosphorothiolated lyophilized oligonucleotides were dissolved in sterile saline solution, fractionated to a final concentration of 50 mg/ml and stored at -20°C. Approximately 30 min before each administration, oligodeoxynucleotides were further dissolved in saline. None of them exhibits homology with other reported sequences in GenBank.

### *In vitro *studies

Studies were carried out using primary cultures from three different tumors (59-2-HI, CC4-HI, and C7-2-HI) [23]. Briefly, trypsinized tumor cells were seeded in multiwell plates; cell proliferation was evaluated 48 hours or 1 week after treatment by [^3^H]thymidine uptake or cell counting, respectively. All experiments were performed with Dulbecco's modified Eagle medium-F12 (Sigma) and steroid stripped fetal bovine serum. Oligodeoxynucleotides designed to block both PR isoforms were used (5'-ACTCATGAGCGGGGACAACA-3') [24]. The scrambled oligonucleotide to PRs (scPR) 5'-ACGCTAGACTGACGACGAGA-3' was used as a control. [^3^H]thymidine uptake index was calculated as experimental counts per min/control counts per min.

The efficiency with which asPR blocked expression of PRs was evaluated in Western blots 24 hours after treatment, using the Ab7 (Neomarkers, Union City, CA, USA) antibody [22] or by binding techniques at single saturating concentrations with [^3^H]R5020, as previously described [23]. NMuMG (murine mammary epithelial cells) [25] and uterus were used as negative and positive controls, respectively.

### *In vivo *experiments

#### Tumor implants

Tumors were implanted subcutaneously into the right inguinal flank by trocar, as previously described [21]. Tumor growth was measured using a Vernier caliper (length and width). The product of these values was considered the tumor area. After the animals were killed, tumors were excised and weighed.

#### Antiprogestin treatment

When the tumors reached a size of approximately 25–50 mm^2^, animals were treated with daily doses of saline, ZK 299 (10 mg/kg), or RU 486 (6.5 mg/kg), as previously described [26]. Selected mice (*n *= 3/group) were killed 24 hours after the first RU 486 injection. In some experiments RU 486 was administered as silastic 5 mg pellets, implanted subcutaneously. Tumor samples were fixed for histological evaluation and others were kept in liquid nitrogen for Western blot studies.

#### Antisense treatment

The treatments were started when the tumors reached 25–50 mm^2^. Two sets of experiments were performed. In the first, animals carrying 59-2-HI or 32-2-HI tumors were injected intraperitoneally with 1 mg phosphorothiolated oligodeoxynucleotides to PR (i.e. asPR; volume 0.2 ml) or saline every 24 and 12 hours (*n *= 3–4/group) for 59-2-HI and 32-2-HI groups, respectively. All injections were prepared in sterile saline immediately before administration. Tumor size was evaluated every day, as described above. The estrous cycle was evaluated using vaginal smears, and the cycle in control animals was compared with those in RU 486-treated and ZK 299-treated animals. The animals were killed after 10 days and complete autopsies performed. Tumors were weighed and samples were immediately frozen or fixed in formalin. PR expression was evaluated using Western blots, as described below.

In the second set of experiments, the experiment (*n *= 4/group) was repeated under the same conditions as described above but with the addition of two further groups: a third group of mice bearing 32-2-HI tumors and treated with scPR; and a fourth group treated with RU 486 in 5 mg pellets implanted subcutaneously. The animals were inoculated every 12 hours with 1 mg asPR and were killed after 5 days of treatment. Two hours before the animals were sacrificed, two animals in every group were injected with 5-bromodeoxyuridine (BrdU; 4 mg/mouse). All samples fixed in formalin were embedded in paraffin using standard protocols, and 5 μm sections were obtained and stained with heamatoxylin–eosin for histological examination.

#### Progesterone receptor binding assays

Binding of [^3^H]R5020 to PRs was evaluated by whole cell assay. Briefly, 10^5 ^cells were plated in 24-well plates with Dulbecco's modified Eagle medium-F12 (Sigma) and 5% steroid stripped fetal bovine serum. Treatments were initiated when cultures were half confluent; whole cell PR assays were performed after 24 hours, as previously described [23]. A total of 300,000 counts/min 17α-methyl-[^3^H]R5020 were added together with a 100-fold excess of R5020 or ethanol. After 2 hours of incubation, the cells were washed, trypsinized, and counted in a liquid scintillation counter. A significant difference between counts per min in the groups incubated only with radioactive hormone and those incubated with radioactive plus unlabeled hormone yields the total counts per min bound to the receptors. These differences are directly proportional to the number of PRs in each experimental group.

### Preparation of whole cell extracts

Uterus, muscle, and tumors were homogenized in a polytron at setting 50 with three bursts of 5 s in a 1:4 ratio of tissue:buffer. The buffer was 20 mmol/l Tris-HCl (pH 7.4), 1.5 mmol/l EDTA, 0.25 mmol/l dithiothreitol, 20 mmol/l Na_2_MoO_4_, and 10% glycerol. Protease inhibitors (0.5 mmol/l phenylmethylsulfonyl fluoride, 0.025 mmol/l N-Carbobenzyloxy-L-phenylalanyl chloromethyl ketone, 0.025 mmol/l tosyl-lysylchloromethane, 0.025 mmol/l tosylphenylalanylchloromethane, and 0.025 mmol/l N_α_-p-Tosyl-L-arginine methyl ester hydrochloride) were added to the buffer immediately before use. The homogenate was sonicated at medium frequency for 10 s (tubes were always kept on ice) and centrifuged for 45 min at 40,000 rpm (4°C). The supernatant was immediately frozen in liquid nitrogen and stored at -70°C until later use in Western blot assays. Protein concentration was determined in accordance with the method proposed by Lowry and coworkers [27].

### Western blot

The samples (100 μg total protein/lane) were separated on 7.5% SDS-PAGE using Laemmli's buffer system [28]. The proteins were dissolved in sample buffer (6 mmol/l (Tris pH 6.8), 2% SDS, 0.002% bromophenolblue, 20% glycerol, 5% mercaptoethanol) and boiled for 4 min. After electrophoresis they were blotted onto a nitrocellulose membrane and blocked overnight in 5% dry skimmed milk dissolved in phosphate-buffered saline (PBS)/Tween 0.1% (0.8% NaCl, 0.02% KCl, 0.144% Na_2_PO_4_, 0.024% KH_2_PO_4_, pH 7.4, 0.1% Tween 20). Following several washes with PBS/Tween, the membranes were incubated with the primary antibody against PRs (Ab-7/hPRa 7 (Neomarkers) or Ab-1 (kindly provided by Dr Gopalan Shyamala)), ER-α (MC-20; Santa Cruz Biotechnology, Santa Cruz, CA, USA), extracellular signal-regulated kinase (ERK; K-23; Santa Cruz Biotechnology), phosphorylated (p)ERK (E-4; Santa Cruz Biotechnology), and E-cadherin (BD Transduction Lab, Palo Alto, CA, USA) at room temperature for 2 hours. Primary antibodies were used at 1:100 concentrations, except for E-cadherin, which was used at 1:10.000. Blots were probed with anti-mouse or anti-rabbit IgG, horseradish peroxidase-conjugated whole antibody (Amersham Life Science). The luminescent signal was generated with ECL Western blotting detection reagent kit (Amersham Pharmacia Biotech, Buckinghamshire, UK), and the blots were exposed to a medical X-ray film (Curix RP1; Agfa, Buenos Aires, Argentina) for 10 s to 5 min. To control the efficiency of transference, membranes were stained with Ponceau S.

### Immunohistochemistry

The sections were de-waxed in xylene, rehydrated through graded ethanols, and treated with 1% triton X-100 in PBS for 20 min at room temperature. They were then washed with PBS three times, 5 min each, and incubated for 30 min at room temperature with 3% H_2_O_2 _in distilled water to quench endogenous peroxidase activity, washed extensively with PBS, and incubated in 3% albumin or normal horse serum in PBS for 20 min. The sections were then allowed to react with PRs (C-20; rabbit polyclonal IgG specific for PR; Santa Cruz Biotechnology) or ER (MC-20; rabbit polyclonal IgG specific for ER; Santa Cruz Biotechnology) diluted 1:100 in PBS for 48 hours at 4°C [25]. The slides were washed with PBS and successively incubated for 30 min at room temperature with anti-rabbit biotin-conjugated immunoglobulins (Vector Labs, San Francisco, CA, USA), diluted 1:250 in PBS, and, with the ABC complex, prepared according to the manufacturer's directions (Vector Labs). The slides were thoroughly washed with PBS and developed under microscopic control with 3-3' diaminobenzidine 0.06% in PBS and H_2_O_2 _at a final concentration of 0.1%. Stained nuclei were counted in 15 high-power fields of each section using a 1000× magnification and expressed as mean ± standard deviation of the percentage of the ratios between the total number of stained nuclei and the total cell number per high-power field.

5-BrdU was detected using a sheep polyclonal antibody (Maine Biotechnology Services Inc., Portland, ME, USA). Briefly, de-waxed and hydrated slides were permeabilized with proteinase K (Sigma; Cat# 92905), 20 μg/ml, 15 min at room temperature. Enzyme action was stopped with 5% normal horse serum in PBS and the slides were successively incubated with the anti-BrdU antibody diluted 1:200 in PBS, overnight at 4°C, and washed and incubated with anti-sheep antibody (Vector) 1:400 in PBS. The slides were then processed through ABC (Vector) and developed as described above. Apoptosis was evaluated using the standard *in situ *cell death detection Kit, Fluorescein (Roche; Cat# 1 684 795), in accordance with the manufacturer's instructions.

### Statistical analysis

Analysis of variance followed by Tukey t-test was used to analyze the differences between control and experimental groups in [^3^H]thymidine uptake, tumor size, PR staining, and binding assays. Unpaired t-tests were used as needed. *P *< 0.05 was considered statistically significant. Values are reported as mean ± standard deviation. Tumor growth curves were also studied using regression analysis and slopes compared using analysis of variance followed by parallelism analysis.

## Results

### *In vitro *studies

**Figure 1:**
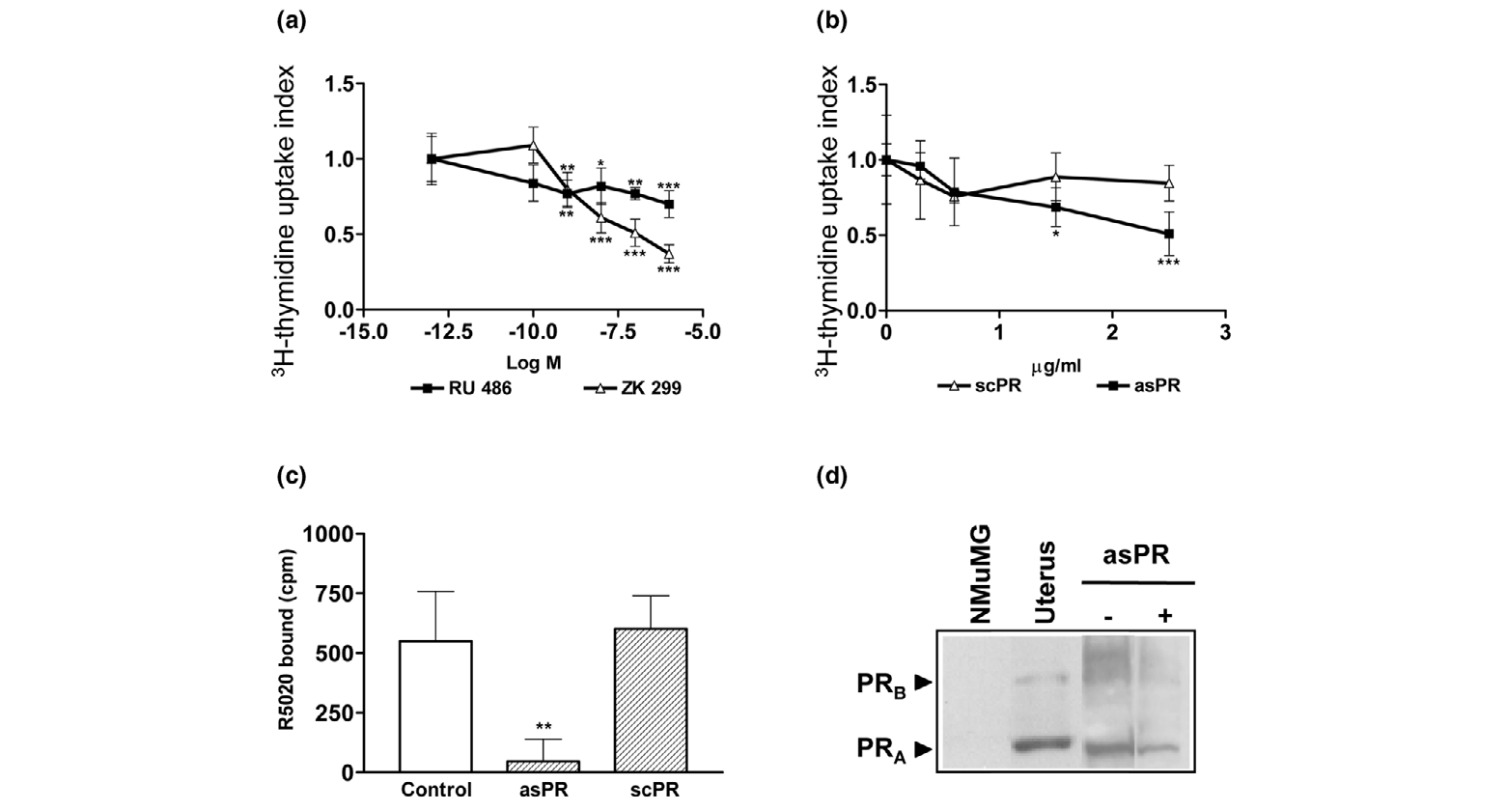
*In vitro *effects of asPR on primary cultures of progestin-independent tumors. Effect of **(a) **antiprogestins (RU 486, ZK 299) or **(b) **oligodeoxynucleotides to PR (asPR or scPR) on [^3^H]thymidine uptake index on primary cultures of responsive progestin-independent tumor cells. The cells were incubated in Dulbecco's modifeid Eagle medium-F12 without phenol red, in the presence of 2.5% ssFCS and different drug concentrations for 48 hours. [^3^H]thymidine was added in the last 18 hours. These are representative experiments from at least three using three different tumors, with each value corresponding to the mean ± SD cpm of octuplicates. [^3^H]thymidine uptake index was calculated as the experimental cpm/control cpm. *P < 0.05, **P < 0.01, ***P < 0.001. **(c) **PR binding was evaluated *in vitro *using the whole cell binding assay in primary cultures from a responsive progestin-independent tumor in the presence of 2.5% ssFCS (control), scPR (5 μg/ml) or asPR (5 μg/ml; **P < 0.01, *P < 0.05). **(d) **PR Western blot analysis of cell extracts from the primary cultures grown in the presence of 2.5% ssFCS (-) or asPR (5 μg/ml). Primary antibody: Ab-7 (mouse monoclonal; Neomarkers). NMuMG cell line and uterus were used as negative and positive controls, respectively. Experimental details are explained in Materials and method. asPR, antisense oligodeoxynucleotides to progesterone receptors; PR, progesterone receptor; scPR, scrambled oligodeoxynucleotides to progesterone receptors; SD, standard deviation; ssFCS, steroid stripped fetal calf serum.

The role played by PRs in progestin-independent tumor growth was investigated *in vitro *using primary cultures of three different tumor cell lines from our model, as previously described [16,23]. RU 486 and ZK 299, two antiprogestins that exert their effects by different mechanisms, were able to inhibit cell proliferation, as evaluated by cell counting (not shown) and [^3^H]thymidine uptake (Fig. 1a), at concentrations as low as 1 × 10^-9 ^mol/l. Using the same experimental conditions, we investigated the effect of asPR. In concentrations above 1.25 μg/ml, inhibition of cell proliferation was observed, as measured by [^3^H]thymidine uptake (Fig. 1b) or by cell counting (control: 64.75 ± 3.84; asPR 5 μg/ml: 38.25 ± 1.14 cells/ml × 10^-4^). No effect was observed in the PR-negative NMuMG cells (not shown) or in tumor cells incubated with scPR (Fig. 1b).

AsPR treatment resulted in potent inhibition of PR binding (*P *< 0.01) whereas scPR had no effect (Fig. 1c). Western blot studies revealed that both PR isoforms were expressed to a lesser degree in asPR-treated cells (Fig. 1d).

### *In vivo *studies

#### Tumor growth

The goal of this study was to extend our *in vitro *findings to an *in vivo *scenario. We showed that the tumors used in this study regressed completely after antiprogestin or estrogen treatment [16]. Our previous *in vivo *data pointed toward an essential role for PRs in tumor growth, but because estrogens induced the same inhibitory effect as antiprogestins, and antiprogestins were shown to exert estrogenic effects [29], the importance of PRs remained to be demonstrated. The aim of the following experiments was to evaluate whether tumor growth could be inhibited by blocking PR expression.

**Figure 2:**
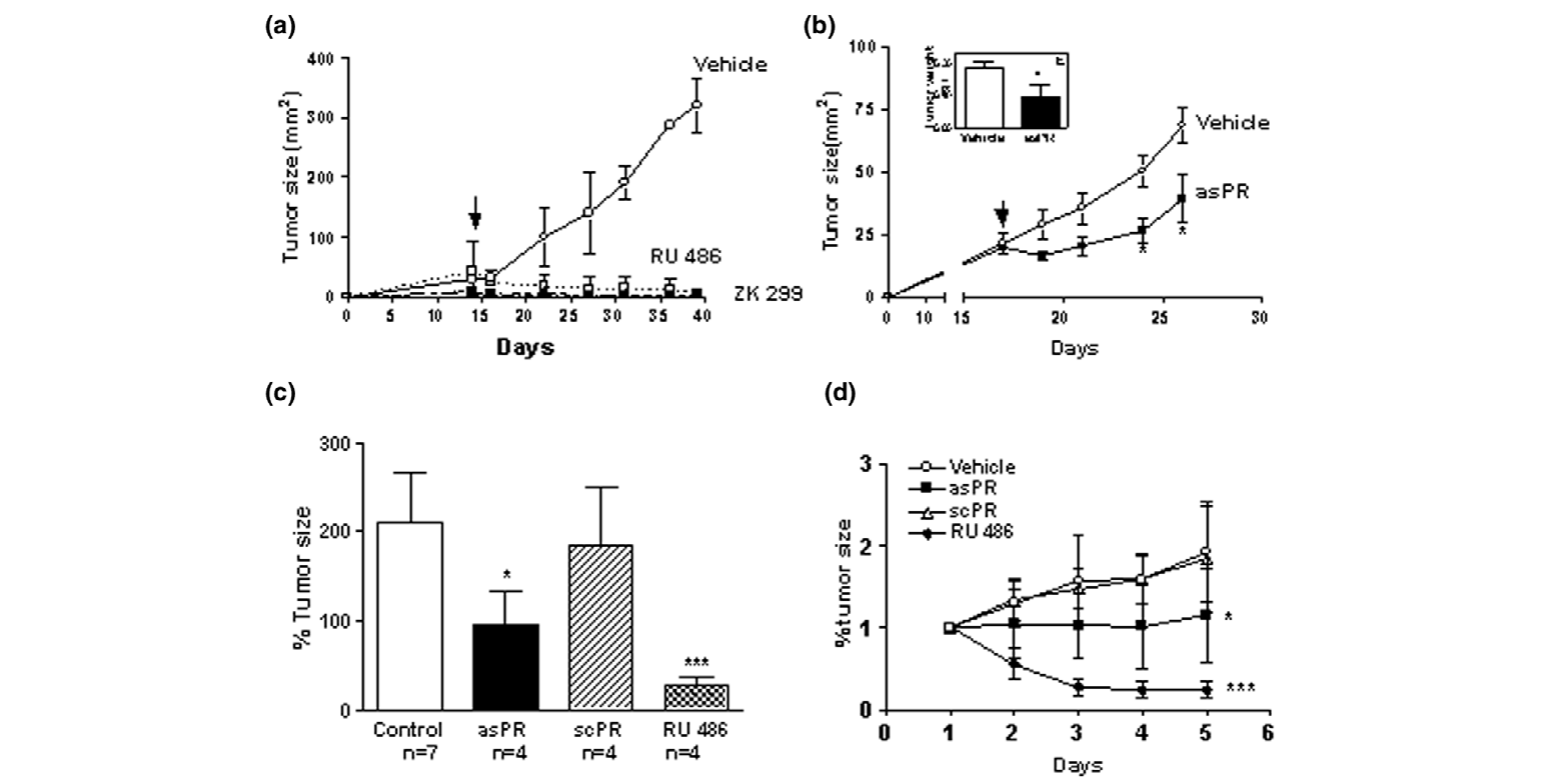
*In vivo *effect of asPR on progestin-independent tumor growth. **(a) **Effect of two different antiprogestins on tumor growth. Animals bearing tumors of size 25–50 mm^2 ^were treated with RU 486 (6.5 mg/kg body weight) or ZK 299 (10 mg/kg body weight subcutaneously) or saline. **(b) **Tumor growth in animals treated with saline (*n *= 7) or asPR (*n *= 3) administered in two daily doses of 1 mg intraperitoneally (*P < 0.05). The differences in tumor growth rate are significantly different (*P *< 0.01). Inset: tumor weight at the end of the experiment, 11 days after treatment was initiated. **(c,d) **Effect of asPR (1 mg/12 hours, intraperitoneally), scPR (1 mg/12 hours, intraperitoneally), or RU 486 (6.5 mg/kg body weight, subcutaneously) on *in vivo *tumor growth. The percentage of the tumor size calculated as the final tumor area/tumor area at the beginning of the experiment (100%) was evaluated on the last day of treatment (panel b) or throughout 5 days of treatment (panel c). asPR, antisense oligodeoxynucleotides to progesterone receptors; scPR, scrambled oligodeoxynucleotides to progesterone receptors. asPR, antisense oligodeoxynucleotides to progesterone receptors; scPR, scrambled oligodeoxynucleotides to progesterone receptors.

In a first set of experiments the progestin-independent tumor line 59-2-HI was chosen because complete regression was induced with RU 486 and ZK 299, even in tumors with an initial size greater than 100 mm^2 ^[26]. In this first experiment, asPR treatment (1 mg/day) began when tumors measured approximately 25 mm^2 ^and continued for 10 days. AsPR inhibited tumor growth (not shown). No signs of nonspecific toxicity were detected by histopathological evaluation of organs. This experiment suggested that, although asPR inhibited tumor growth, the appropriate antisense dose could still be increased. Consequently, a second set of experiments was carried out using the 32-2-HI tumor, which also regresses completely after antiprogestin treatment (Fig. 2a), and the asPR dose was increased to 1 mg twice daily. Figure 2b shows that asPR treatment significantly inhibited tumor growth for 5 days, after which tumors resumed growth but at a slower rate.

From these experiments it was evident that asPR inhibited tumor growth. In a third set of experiments we included the control group using a scrambled sequence (scPR) and focused on short-term effects of asPR. In addition to tumor size, histopathological studies of tumors during the stationary phase were performed. As shown in Fig. 2 (panels c and d) the differences in tumor size between asPR-treated animals and vehicle-treated or scPR-treated mice were significant. However, a more conspicuous decrease in tumor size was observed in RU 486-treated animals.

### Estrous cycle

Vaginal smears were evaluated daily in RU 486-, ZK 299-, and asPR-treated animals. During the first week of treatment, AsPR-treated mice and antiprogestin-treated mice were in a continuous estrous/meta-estrous state; this effect of antiprogestins on the estrous cycle has been described by others [30,31]. After 1 week of treatment, asPR-treated mice started to cycle again, and interestingly tumors started to grow again. This transient effect of asPR treatment on the estrous cycle parallels the effect observed on tumor growth. E_2 _serum levels in asPR-treated animals were undetectable by radioimmunoassay, whereas those in control animals were in the expected range (not shown). Thus, the estrous state was not achieved because of higher E_2 _serum levels.

### Histopathology

**Figure 3:**
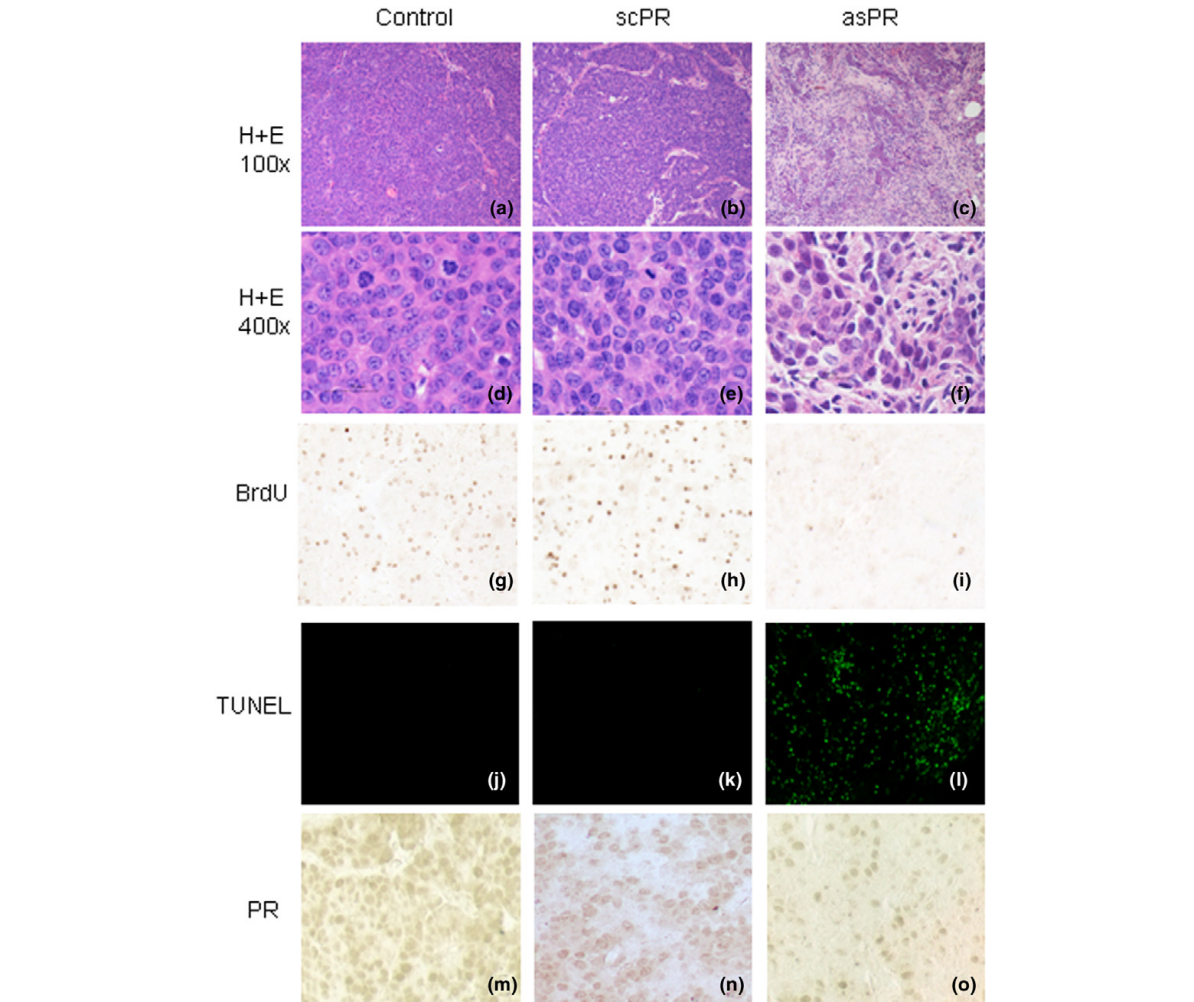
Morphological features and PR immunostaining in tumors treated with asPR or scPR for 5 days. Left column: control mice treated with saline; middle column: mice treated with scPR; right column: mice treated with asPR. **(a–c) **Hematoxylin and eosin (100×); **(d-f) **Hematoxylin and eosin (400×); **(g–i) **5-BrdU immunocytochemistry; **(j–l) **TUNEL (100×); **(m-o) **PR immunohystochemistry. No changes were observed in tumors from animals treated with saline or scPR. Tumors from animals treated with asPR exhibit increasing degrees of fibrosis, as well as occasional lymphocytic infiltration. TUNEL staining, indicative of apoptosis, was only observed in asPR-treated tumors (panel i), in which also very few 5-BrdU stained nuclei were observed (panel f). Few PR stained nuclei are observed in asPR-treated tumors (panel l) as compared with control or scPR-treated tumors, in which most of the cells are positive (panels j and k, respectively). asPR, antisense oligodeoxynucleotides to progesterone receptors; PR, progesterone receptor; scPR, scrambled oligodeoxynucleotides to progesterone receptors; TUNEL, terminal deoxynucleotidyl transferase mediated dUTP nick-end labeling.

Tumor regression in this model progresses through cytostasis and apoptosis, leading to a progressive reduction in the epithelial compartment accompanied by an increase in stroma [26]. Histological signs of regression similar to those observed in the RU 486-treated tumors were seen in areas of asPR-treated mice (Fig. 3c,f) but not in vehicle-treated (Fig. 3a,d) and scPR-treated animals (Fig. 3b,e). The lesions revealed the presence of fibrosis; in areas the tumor was reduced to few strands of epithelial cells and occasional areas of calcification were observed. Althugh BrdU staining was similar between control and scPR-treated animals, absence of BrdU labeling was observed in one tumor (1/4) and a decrease in the other three tumors of asPR-treated mice (Fig. 3g–i). A greater number of apoptotic cells, as shown by TUNEL (terminal deoxynucleotidyl transferase mediated dUTP nick-end labeling) assay, were observed in regressing tumors (Fig. 3j–l).

**Figure 4:**
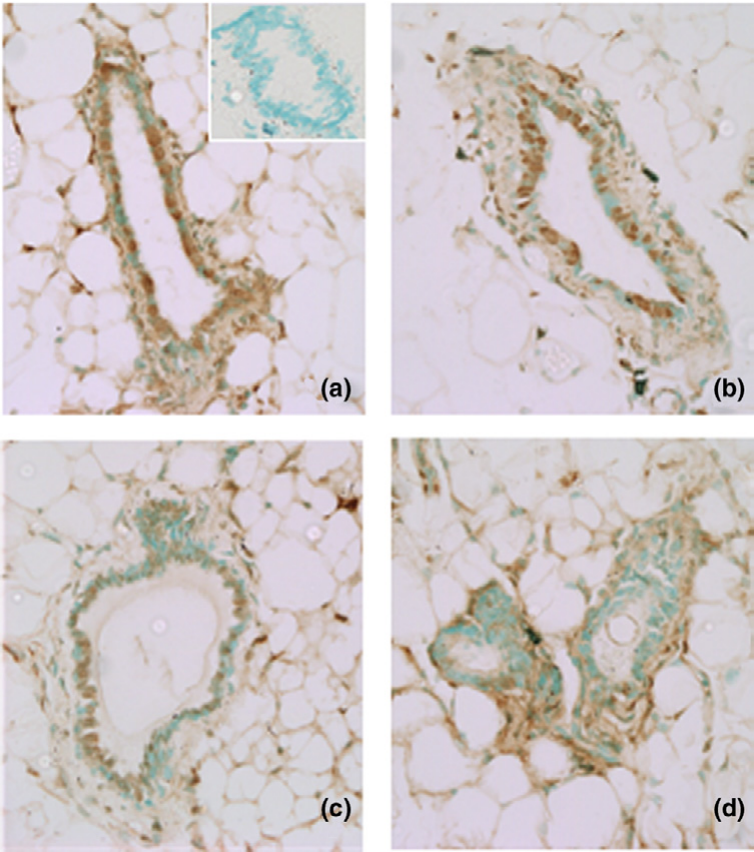
Effects of asPR or RU 486 on PR expression in mammary glands. Immunohistochemical staining for PR in sections of paraffin-embedded mammary glands of mice treated with **(a) **saline, **(b) **scPR, **(c) **asPR, or **(d) **RU 486 for 5 days. Inset: control, in which no primary antibody was added. Primary antibody: PR polyclonal (C-20; Santa Cruz Biotechnology). The procedure was performed as described in Materials and method. asPR, antisense oligodeoxynucleotides to progesterone receptors; PR, progesterone receptor; scPR, scrambled oligodeoxynucleotides to progesterone receptors.

To confirm the efficiency of asPR treatment in blocking expression of PRs, they were evaluated by immunohistochemistry in tumor samples as well as in normal mammary glands from treated and untreated mice (Fig. 3m–o). A significant decrease (*P *< 0.01) in PR staining was observed in tumors from asPR-treated mice as compared with tumors from control and scPR-treated mice. The percentage of stained nuclei was evaluated in 45 high-power fields from three different tumors (mean ± standard deviation; control: 46 ± 6.4%; scPR: 44.7 ± 3%; asPR: 11 ± 1.3%). No staining was observed using these antibodies in mammary glands from PR knockout mice [22]. Interestingly, no differences in PR (Fig. 4a–c) staining were observed in the mammary glands from asPR-treated, scPR-treated, or control mice, but glands from RU 486-treated mice exhibited only isolated PR immunoreactivity (Fig. 4d). These results suggest that asPR may be more easily captured by tumor cells than by normal quiescent cells. Histopathological evaluation of lungs, kidney, liver, and spleen revealed no differences among groups (data not shown).

### PR, ER-α, and ERK expression in RU 486-treated and asPR-treated tumors

**Figure 5:**
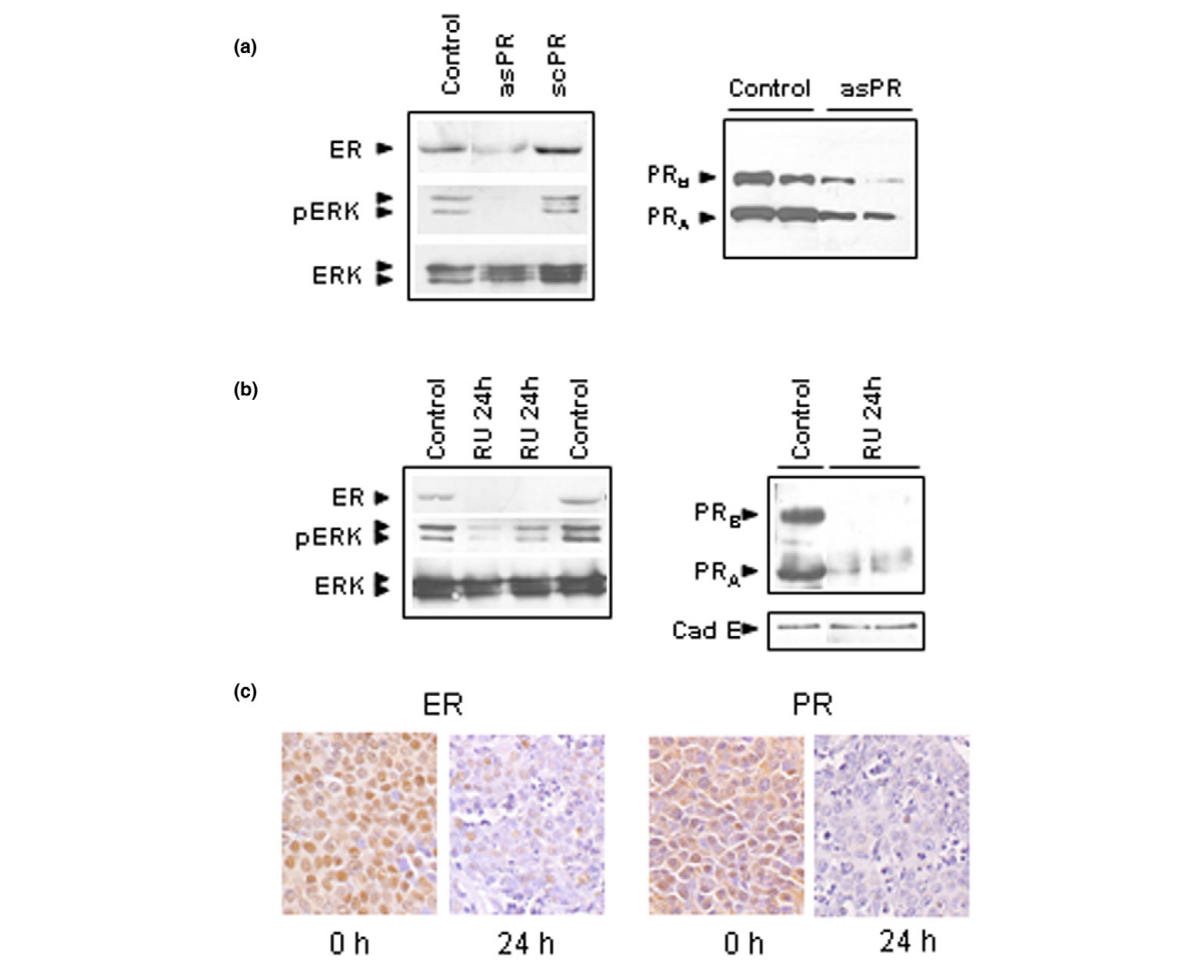
Effects of asPR or RU 486 on MAPK phosphorylation and on ER-α and PR expression. **(a) **Immunoblots of ER-α (MC-20; Santa Cruz Biotechnology), total ERK (K-23; Santa Cruz Biotechnology), and pERK (E-4; Santa Cruz Biotechnology) in whole extracts of tumors obtained from animals treated with saline, asPR, or scPR for 5 days. Tumor samples were obtained after day 5 of treatment; tumor growth kinetics is shown in Fig. 2d. Arrows show ERK1 (42 kDa) and ERK2 (44 kDa). PR immunoblots were performed using extracts obtained from mice treated with asPR over 10 days (Ab1; Dr Shyamala). Arrows show the classical PR_B _of 115 kDa and the classical PR_A _of 83 kDa. **(b) **Immunoblots of ER-α, PR (Ab7; Neomarkers), E-cadherin (E cad; BD Transduction Lab), total ERK, and pERK using wholeextracts of tumors obtained from animals treated with saline or RU 486 for 24 hours. Tumor kinetics are shown in Fig. 2c. A representative Western blot of three is shown. **(c) **Immunohistochemistry of ER-α and PR (C-20 Santa Cruz) of the same tumor samples used in Western blot studies shown in panel b (125×). Experimental details are described in Materials and method. asPR, antisense oligodeoxynucleotides to progesterone receptors; ER, estrogen receptor; ERK, extracellular signal-regulated kinase; MAPK, mitogen-activated protein kinase; PR, progesterone receptor; scPR, scrambled oligodeoxynucleotides to progesterone receptors.

To further compare asPR and RU 486 treatments, we evaluated the expression of ER-α and ERKs in the same asPR-treated samples and in tumors treated with RU 486 for 24 hours. ER-α and pERK1 and pERK2 were decreased in both asPR-treated (Fig. 5a) and RU 486-treated tumors (Fig. 5b), whereas total MAPK levels remained unchanged. PR expression was also down-regulated in asPR-treated tumors (Fig. 5a), as previously shown by immunohistochemistry (Fig. 3m–o), and in the 24-hour RU 486-treated tumors (Fig. 5b). To confirm that this downregulation was not an artifact associated with a decrease in epithelial cells, the expression of E-cadherin – a specific marker of epithelial cells – was used to evaluate the same control and 24-hour RU 486-treated tumor samples. As shown in Fig. 5b, the low levels in steroid receptor expression were not due to differences in the ratio of the epithelial cells. In addition, as shown in Fig. 5c, immunostaining of ER-α and PRs confirmed Western blot data.

## Discussion

During the past few years the PR pathway has emerged as a likely player in the pathogenesis of breast cancer, with growing experimental as well as clinical evidence pointing to its protagonistic role [15]. Although estrogens remain the main foes in this story, interestingly most of the epidemiological evidence for their purported role as mammary carcinogens reflects prolonged exposure to female hormones, including progesterone. Among the abundance of data available, the results from the Women's Health Initiative [1] and the Million Women Study [2] are especially striking because they specifically link the use of progestins with breast cancer. Taking into account the literature attesting to the proliferative effect of progesterone on the mammary gland and the carcinogenic effect of MPA, it may be suggested that these results were predictable [3,4,32].

In this report, using an experimental model of mammary cancer, we demonstrate that the PR pathway is essential to maintenance of cell proliferation, even in tumors that are no longer dependent on progestins to grow. Even though our results do not provide additional data regarding the mechanisms underlying the role played by PRs in mediating tumor growth, they provide further support to our hypothesis and extend our previous data to the whole category of progestin-independent tumors. This experimental model shares many features with human breast cancer, in that the tumors are ductal metastatic carcinomas expressing high levels of ER-α and PR, which transit through different stages of hormone dependency. In addition, tumors regress completely after antiprogestin or estrogen therapy and partially with tamoxifen [33]. The effectiveness of antiprogestins in tumor models other than ours, such as the rat MNU model or the mouse MXT model, has also been addressed by other authors [34].

There are no data available regarding the use of PRs as targets for gene therapy in breast cancer. Considering all of our previous findings, it seemed mandatory to try to inhibit the expression of PRs and to demonstrate their role in an *in vivo *scenario. Because we were working with tumors and not with cell lines, we chose naked phosphorothiolated antisense oligonucleotides to PR. Using this approach, several genes such as *ras*, *myc*, *mib*, *mdm-2 *and *bcr-abl*, genes related to apoptotic functions such as *bcl-2*, and genes related to multidrug resistance have been blocked [35]. In all cases only incomplete blockade of protein expression was observed, and so only slight changes in tumor growth rate were evident. The results improved when combined therapies were applied [36].

In our experiments we achieved significant inhibition of tumor growth using one or two daily doses of 1 mg asPR as compared with control or scPR-treated mice. No signs of toxicity were found at autopsy. Safety studies conducted in experimental animals have shown that repeated administration of phosphorothiolated oligonucleotides containing CpG dinucleotide motifs in a particular base context or G quartets provoke adverse side effects due to cytokine release, decreased platelet counts and hepatotoxicity resulting from nonspecific immune stimulation [37]. The oligonucleotides used herein do not have CpG motifs but they do bear a G quartet; however, no signs of nonspecific immune reaction were observed on histological examination. The inhibitory effect on tumor growth correlated with a decrease in PR expression, but PR blockade was incomplete. This is in agreement with the partial inhibition of tumor growth and with results obtained by other groups using different antisense oligonucleotides [36,38]. It is also interesting that PR expression in mammary glands from asPR-treated mice was not affected to the degree that PRs from tumors were. The difference in effectiveness of the antisense therapy between tumors and normal tissues is an issue that has not been addressed in most studies [39-41]; on the other hand, it has been shown in *in vitro *studies that antisense treatment is less effective in normal cells than in the tumor cells [42]. From these observations it can be inferred that oligonucleotides may be more easily captured in a tumor environment than by quiescent cells. Although preliminary data obtained in our laboratory suggests that, in tumors implanted subcutaneously, there is an increased uptake of ^32^P-labeled oligonucleotides as compared with the normal mammary gland (not shown), this may be different in spontaneously arising tumors, which have a slower growth rate and abundant stroma.

Antiprogestins induce a continuous estrous or meta-estrous state [30]. Estrogen-like activities have been demonstrated for both antiprogestins, RU 486 [29], and ZK 299 [31,43] in both *in vivo *and *in vitro *studies. There has been concern from an endocrinological perspective over whether this effect was achieved because of a direct interaction of antiprogestins with ER-α. The continuous estrus/meta-estrus state also observed in asPR-treated mice favors the notion that unopposed low estrogen levels may be responsible for inducing this uterotrophic effect [44]. These low E_2 _levels are not the cause of tumor regression because these tumors grow similarly in ovariectomized animals.

Because ERK phosphorylation is among the signal transduction pathways involved in steroid-induced cell proliferation [45], we explored the expression of these proteins in RU 486-treated and asPR-treated mice. An important decrease in pERK1 and pERK2 levels was evident after 24 hours of treatment when the tumors had already experienced a decrease in size. Similarly, in asPR-treated mice undergoing tumor regression, a decrease in pERK was observed, once again showing parallelism between RU 486-induced and asPR-induced tumor regression. ER-α expression exhibited the same kinetics. Absence of ER-α expression was observed in asPR-treated tumors and in RU 486-treated tumors after 24 hours. Immunohistochemistry studies confirmed results observed by Western blots ruling out the possibility that, although the same amount of protein was seeded, fewer epithelial cells expressing high levels of ER-α were masked.

## Conclusion

Our findings provide the first evidence that blockade of PRs using antisense oligonucleotides induces inhibition of tumor growth, and provide further evidence for a critical involvement of the stimulatory effects of the PR pathway in mammary cancer, supporting its choice as an alternative therapeutic target for those tumors bearing receptors that are unable to bind ligands.

## Abbreviations

asPR = antisense oligodeoxynucleotides to progesterone receptors; BrdU = 5-bromodeoxyuridine; E_2 _= estradiol; ER = estrogen receptor; ERK = extracellular signal-regulated kinase; MAPK = mitogen-activated protein kinase; MPA = medroxyprogesterone acetate; PBS = phosphate-buffered saline; PR = progesterone receptor; scPR = scrambled oligodeoxynucleotides to progesterone receptors.

## Competing interests

The authors declare that they have no competing interests.

## Authors' contributions

CAL carried out all of the *in vitro *experiments and, together with LAH, participated in the *in vivo *experiments. SG and LAH carried out the Western blots assays. RS and SV participated in all immunohistochemical studies. CL and AM designed, coordinated, and drafted the manuscript. All authors read and approved the final manuscript.
